# Cholesteryl Ester Transfer Protein Inhibitors in the Treatment of Dyslipidemia: A Systematic Review and Meta-Analysis

**DOI:** 10.1371/journal.pone.0077049

**Published:** 2013-10-28

**Authors:** Chuanwei Li, Wen Zhang, Faying Zhou, Caiyu Chen, Liang Zhou, Yafei Li, Ling Liu, Fang Pei, Hao Luo, Zhangxue Hu, Jing Cai, Chunyu Zeng

**Affiliations:** 1 Department of Cardiology, Daping Hospital, The Third Military Medical University, Chongqing, PR China; 2 Chongqing Institute of Cardiology, Chongqing, PR China; 3 Department of Respiratory, Xinqiao Hospital, The Third Military Medical University, Chongqing, PR China; 4 Department of Health Statistics, College of Preventive Medicine, Third Military Medical University, Chongqing, PR China; 5 Department of Social Medicine and Health Service Management, College of Preventive Medicine, Key Lab of Medical Protection for Electromagnetic Radiation, Ministry of Education of China, Third Military Medical University, Chongqing, PR China; FuWai hospital, Chinese Academy of Medical Sciences, China

## Abstract

Cholesteryl ester transfer protein (CETP) inhibitors are gaining substantial research interest for raising high density lipoprotein cholesterol levels. The aim of the research was to estimate the efficacy and safety of cholesteryl ester transfer protein inhibitors as novel lipid modifying drugs. Systematic searches of English literature for randomized controlled trials (RCT) were collected from MEDLINE, EBASE, CENTRAL and references listed in eligible studies. Two independent authors assessed the search results and only included the double-blind RCTs by using cholesteryl ester transfer protein inhibitors as exclusively or co-administrated with statin therapy irrespective of gender in enrolled adult subjects. Two independent authors extracted the data by using predefined data fields. Of 503 studies identified, 14 studies met the inclusion criteria, and 12 studies were included into the final meta-analysis. Our meta-analysis revealed that CETP inhibitors increased the HDL-c levels (n = 2826, p<0.00001, mean difference (MD)  = 20.47, 95% CI [19.80 to 21.15]) and total cholesterol (n = 3423, p = 0.0002, MD = 3.57, 95%CI [1.69 to 5.44] to some extent combined with a reduction in triglyceride (n = 3739, p<0.00001, MD = −10.47, 95% CI [−11.91 to −9.03]) and LDL-c (n = 3159, p<0.00001, MD = −17.12, 95% CI [−18.87 to −15.36]) irrespective of mono-therapy or co-administration with statins. Subgroup analysis suggested that the lipid modifying effects varied according to the four currently available CETP inhibitors. CETP inhibitor therapy did not increase the adverse events when compared with control. However, we observed a slight increase in blood pressure (SBP, n = 2384, p<0.00001, MD = 2.73, 95% CI [2.14 to 3.31], DBP, n = 2384, p<0.00001, MD = 1.16, 95% CI [0.73 to 1.60]) after CETP inhibitor treatment, which were mainly ascribed to the torcetrapib treatment subgroup. CETP inhibitors therapy is associated with significant increase in HDL-c and decrease in triglyceride and LDL-c with satisfactory safety and tolerability in patients with dyslipidemia. However, the side-effect on blood pressure deserves more consideration in future studies.

## Introduction

Cardiovascular disease(CVD)remains to be the leading cause of mortality and morbidity worldwide despite numerous therapeutic advances and steady decline in mortality in recent years [1]. Statin therapy is the cornerstone of pharmacological therapy in both the primary and secondary prevention and has been demonstrated in a series of randomized control trials [2]. It is estimated that lower total cholesterol levels accounts for about 24% reduction in coronary heart disease deaths [3]. However, the CVD mortality remains high in spite of intensive cholesterol lowering therapy to reduce the low density lipoprotein cholesterol (LDL-c) to 100 mg/dl or lower [4].

Aside from LDL-c, high density lipoprotein (HDL) is an attractive target for CVD therapy to further reduce the residual risk from cardiovascular events. HDL cholesterol (HDL-c) level has been found to be inversely correlated with CVD morbidity. It was estimated that a 1 mg/dl increment in HDL-c was associated with a 2–3% reduction in the risk from cardiovascular disease [5]. Even in statin treated patients, low HDL-c levels remains to be significantly and independently associated with increased cardiovascular risk [6]. To date, two HDL-c elevating drug classes, fibrates and niacin are currently used in clinical applications. They can effectively increase the HDL-c range from 10% to 16% with a 20–36% reduction in triglyceride levels. However, the beneficial effects on mortality are limited [7]. Therefore, a new kind of medicine to increase HDL-c levels is needed as an alternative method to increase HDL-c and finally reduce CVDs.

Cholesteryl ester transfer protein mediates the bidirectional transfer of neutral lipids between the triglyceride rich lipoproteins and HDL. Mice are naturally CETP deficient and exhibit relative resistance to a high-fat diet induced atherosclerosis. Meanwhile transgenic exogenous CETP expression in apolipoprotein E (apoE) or LDL receptor knock-out mice exhibit an increased susceptibility to arterial atherosclerosis [8]. Plasma CETP mass and activity are elevated in CVD patients or those with high CVD risk, resulting in decreased HDL and increased triglycerides (TG). CETP quantity and activity also reflect atherosclerosis status. Some pilot studies have revealed a positive correlation between the carotid *intima media* thickness (IMT) and CETP concentration [9]–[10]. Three single nucleotide polymorphisms in the CETP gene are associated with decreased CETP activity and elevated HDL-c levels in carriers and inversely related with coronary risk, making CETP inhibitors reasonable HDL-c based therapeutic agents [11]–[12]. In rabbit models, the CETP inhibitor JTT-705 form a disulphide bond with CETP to down-regulate more than 70% of CETP activities, resulting in a 35% increase in HDL-c and inhibit the progression of atherosclerosis [13]. CETP inhibitors comprise of a drug class which, includes: torcetrapib, dalcetrapib (JTT-705), anacetrapib, evacetrapib. They could inhibit CETP activity and thus increase the formation of high density lipoprotein levels in various degrees. There are some early clinical trials showing the inspiring results of CETP inhibitors in the treatment of patients with dyslipidemia [14]–[25]. However, negative or opposite results were also reported in some clinical trials. For example, Hermannn [14] reported a slightly increase in TC (19.3 mg) after 600 mg dalcetrapib treatment, while de Grooth [16] failed to find any change in TC irrespective of the dose. Moreover, the effects of individual CETP inhibitors vary. The reasons leading to the differences are not known, but might be related with the study design, treatment duration, drug dosage, and other factors. However, CETP inhibitors still remain an important therapeutic option for further reducing the residual CVD risk by targeting HDL. We performed a meta-analysis of all published randomized controlled trials by using CETP inhibitors as a mono-therapy or co-administered with statins versus placebo for treating patients with dyslipidemia. As most of the treatment durations of the enrolled studies are relatively short (4–12 weeks), we mainly focused on the lipid modifying efficacy and safety of CETP inhibitors in patients with dyslipidemia.

## Methods

### Data source, search strategy, and selection criteria

The meta-analysis was performed according to The PRISMA statement for reporting systematic reviews and the latest Cochrane handbook for systematic reviews of intervention (Version 5.1.0, 2011) [26]. Studies were included by searching literatures from MEDLINE, EBASE and Cochrane controlled clinical trails register (CENTRAL) using the key words and references listed in eligible studies from 1965 to April 12, 2012. The following key words were used as highly sensitive search strategy in MEDLINE and modified to apply to the other databases: Torcetrapib* OR Dalcetrapib* OR Anacetrapib* OR Evacetrapib* AND randomized controlled trial OR controlled clinical trial OR randomized OR placebo OR drug therapy OR randomly OR trial OR groups NOT animals NOT humans. The search was restricted to papers published in English, conducted on human subjects and classified as RCTs. Original studies were included if they met the following criteria: (1) RCTs using CETP inhibitors in treating patients with dyslipidemia; (2) lipid levels at baseline and after treatment or net changes after the treatment; (3) treatment duration longer than 4 weeks. Retrospective studies, observational studies, case studies, and studies with a crossover design were excluded.

### Data extraction and quality assessment

Two independent authors (Li C, Zhang W) extracted the data after fully reading the contents of the final set of included studies by using a predefined data field. One author first extracted the data which was then checked by the other. Disagreements were resolved by discussions between the two authors. If no consensus was achieved, the corresponding author would assess opinions from both sides and make the final decision. The predefined data field included information regarding inclusion criteria, risk of bias, clinical outcome and adverse events. If a trial is reported at several time points, we included the last reported follow-up point. We contacted the authors of enrolled studies for clarification regarding missing data and issues of risk of bias assessment. We assessed the risk of bias of included studies based on the following criteria: sequence generation, allocation concealment, blinding of participants and personnel, blinding of outcome assessment, reporting of incomplete outcome, and other bias. The intention to treat the analysis independently was undertaken, and the last observation was carried forward as the method adopted to deal with missing values. We evaluated the quality of the included studies using the 5 point Jadad score, which provided the basis of randomization, concealment of treatment allocation, blinding, completeness of follow-up, and use of intention-to-treat analysis.

### Data synthesis and statistical analysis

Quantitative variables are expressed as mean±standard deviation (SD), while qualitative variables are expressed as raw numbers and percentages. If some data were not listed on the papers, authors were contacted to obtain the missing data. The data needed to measure the weighted mean difference include: (1) the mean absolute change of lipoproteins and apolipoprotein levels (TC, TG, HDL-c, LDL-c, apoB-100, apo-AI) from baseline to the longest follow up time in milligrams per deciliter (mg/dl). (2) Systolic Blood Pressure (SBP), Diastolic Blood Pressure (DBP) in millimeters of mercury (mmHg). When TC, HDL-c and LDL-c are expressed by mmol/l, multiply by 38.6 to convert to mg/dl, TG is converted to mg/dl by multiplying by 88.5. If the results are expressed by median and range, the mean and standard deviation were calculated according to Hozo and Liu's methods [27]–[28]. Treatment groups with multiple doses were combined to create a single pair-wise comparison with the primary comparisons being treatment versus placebo. We calculated the average mean and SD of multiple dose response groups by the following formula [29]:

m  =  mean, sd  =  standard deviation, i  =  intervention

1. Mean: 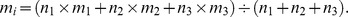

2. Standard Deviation:
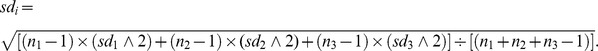

Primary efficacy outcomes were calculated as the net change in lipid and apolipoprotein levels between baseline and longest follow up in response to CETP inhibitor therapy. The secondary efficacy outcomes included the HDL subclasses (HDL_2_, HDL_3_). The safety outcomes comprised of clinical and laboratory adverse events. The laboratory adverse events were: hepato-toxicity (defined as 3 folds higher than the upper limit of normal serum alanine aminotransferase and aspartate aminotransferase levels), musculoskeletal injury (defined as 5 folds higher than the upper limit of normal creatine phosphokinase value). The clinical adverse events were comprised of drug associated adverse events and withdrawals including the mean changes in SBP and DBP after CETP treatment. If the authors did not list the mean differences but provide the mean and/or SD instead, we calculated the mean differences from the other studies in this review by the following formula according to the latest version of Cochrane's handbook for systematic reviews.
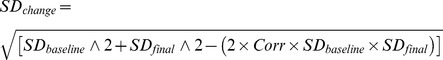


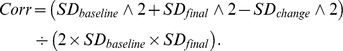



### Assessment heterogeneity and publishing bias in included studies

Heterogeneity between the trials was evaluated by the Cochrane Q test and the magnitude of heterogeneity was assessed by I^2^ statistics. I^2^≥50% was considered to be representative of high heterogeneity. When apparent heterogeneity was observed, we compared performing subgroups by based on the summary of results grouped by age, CETP inhibitor used, dose and duration of treatment, dyslipidemia types, mono-therapy or co-administration with statins, baseline lipid levels and study quality to identify the perceived potential. Sensitivity analysis was performed by repeating the analysis then subsequently removing 1 study group each time. The meta-analysis was performed by Review Manager (REVMAN) software, Version 5.1(The Cochrane Collaboration, Nordic Cochrane center, Copenghagen, Denmark). A fixed effect model was selected preferentially except those where unexplained statistical heterogeneity was identified. Visual inspection of the funnel plot was used to detect the presence of publication bias. We also assayed the possibility of publication bias using the Egger's regression test.

## Results

### Description of included studies

A total of 503 records were obtained by our electronic search, of which 465 records were excluded after screening the titles and abstracts. Two independent authors read the full articles and additional 24 articles were excluded for the reasons listed in the study flow chart (**Figure S1**). Fourteen articles matched our inclusion criteria. However, two more studies with repeated reports in different published papers were further excluded. We finally included 12 researches into the final meta-analysis (n = 2928). Characteristics of the included studies are presented in (**Table 1**). The included studies involved four kinds of CETP inhibitors (Dalcetrapib = 5, Torcetrapib = 4, Anacetrapib = 2, Evacetrapib = 1). 5 studies used CETP inhibitors as mono-therapy and another 5 studies were co-administered with statins. Bloomfield's [23] and Nicholls' [25] studies involved two interventions using CETP inhibitors either as mono-therapy or co-administered with statins. We included them as pair-wise comparisons into the meta-analysis as two independent studies. Most of the treatment durations ranged from 4 weeks to 16 weeks except for Vergeer's [21] study, which was a pooled analysis of the Rating Atherosclerotic Disease Change by Imaging With a New CETP Inhibitor (RADIANCE) Trials 1 and 2, and had a treatment duration of as long as 2 years. The summary of risk of bias of included studies is listed visually in **Figure S2**. The quality of the included trials was evaluated by Jadad score. Overall, three of the included studies scored 5 [23]–[25], one scored 4 [21], seven scored 3 [14]–[20], and the remaining one scored 2 [22]. We did not detect the publishing bias by Egger's test (HDL-c, p = 0.107, 95% CI [−9.56 to 1.14]; TC, p = 0.297, 95% CI [−1.29 to 3.85]; LDL-c, p = 0.499, 95% CI [−2.49 to 4.78]; TG, p = 0.235, 95% CI [−0.43 to 1.59]).

**Table 1:** Characteristics of the included studies*.

Study	Area	Design	Diagnosis	Other therapy	Control	Dose (mg)	Duration (weeks)	Number	mean age (year)	Lipid parameters
**Dalcetrapib**										
**Hermannn, 2009** [**14**]	Zurich	R,DB,PC,P	Type II hyperlipidemia	None	Placebo	600	4	18	58±3.89	TC,TG, LDL-c,HDL-c
**Ballantyne, 2012** [**15**]	USA	R,DB,PC,P#	HDL-c <40 mg/dl or average<50mg/dl	P40	Placebo	300,600, 900	12	292	56.70±10.50	TG,LDL-c, HDL-c,apoAI
**de Grooth, 2002** [**16**]	Multicenter	R,DB,PC,P	Mild hyperlipidemia	None	Placebo	300,600, 900	4	198	50.82±10.13	TC,TG, LDL-c,HDL-c,apoAI,apoB
**Kuivenhoven,2005** [**17**]	Multicenter	R,DB,PC,P#	Type II dyslipidemia	P40	P40	400,600	16	152	54±7.95	TC,TG, LDL-c,HDL-c,apoAI,apoB
**Bisoendial, 2005** [**18**]	Dutch	R,DB,PC,CO	Family hypoalphalipoproteinemia	None	Placebo	600	4	38	42.9±13.9	TC,TG, LDL-c,HDL-c,apoAI
**Torcetrapib**										
**Davidson, 2006** [**19**]	Multicenter	R,DB,PC,P	Below average HDL-c	None		10,30,60, 90	8	162	46.89±10.12	TC,TG, LDL-c,HDL-c,apoAI,apoB
**Mckenndy, 2006** [**20**]	Multicenter	R,DB,PC,P#	Below average HDL-c, eligible for statin	At20	At20	10,30,60, 90	8	174	49.21±8.55	TC,TG,LDL-c,HDL-c,apoAI,apoB
**Vergeer, 2008** [**21**]	Multicenter	R,DB,PC,P#	Heterozygous family hypercholesterolemia and mix dyslipidemia	At- titrated	At- titrated	60	2 years	827	51.8±11.9	TC,TG, LDL-c,HDL-c
**Guerin, 2008** [**22**]	France	R,O,PC,CO#	Type IIB hyperlipidemia	At10	At10	60	6	36	46±7	TC,TG,LDL-c,HDL-c,apoAI
**Anacetrapib**										
**Bloomfield, 2009** [**23**]	Multicenter	R,DB,PC,P	Primary or mixed hyperlipidemia	None	Placebo	10, 40, 150, 300	8	294	56.4±9.6	TC,TG, apoAI,apoB
		R,DB,PC,P§	Primary or mixed hyperlipidemia	At20	At20	10, 40, 150, 300	8	295	56.2±10.72	TC,TG, apoAI,apoB
**Krishna, 2007** [**24**]	USA	R,DB,PC,P	Dyslipidemia and mixed hyperhcholesterolaemia	None	Placebo	10, 40, 150, 300	4	49	_	TC,TG, LDL-c,HDL-c apoAI,apoB
**Evacetrapib**										
**Nicholls, 2011** [**25**]	Multicenter	R,DB,PC,P	Low HDL-c or high LDL-c and TG<400 mg/dl	None	Placebo	30,100, 500	12	156	57.77±10.72	TC,TG, LDL-c,HDL-c,apoAI,apoB
	Multicenter	R,DB,PC,P§	Low HDL-c or high LDL-c and TG<400 mg/dl	At20 or S40 or R10	At20 or S40 or R10	30,100, 500	12	237	58.71±10.72	TC,TG, LDL-c,HDL-c,apoAI,apoB

* Values are presented as mean ± SD ^§^ co-administrated with statin without statin run-in period.

# co-administrated with statins with statin run-in period.

R = randomized; DB = double blind; PC =  placebo controlled; P = parallel; CO =  crossover; P40 = pravastatin 40 mg; At20 = atorvastatin 20 mg; At10 = atorvastatin 10 mg; S40 = smivastatin 40 mg; R10 = Rosuvastatin 10 mg; At-titrated = atorvastatin titrated to target LDL-c.

### Lipid modifying effects

**Figure 1:**
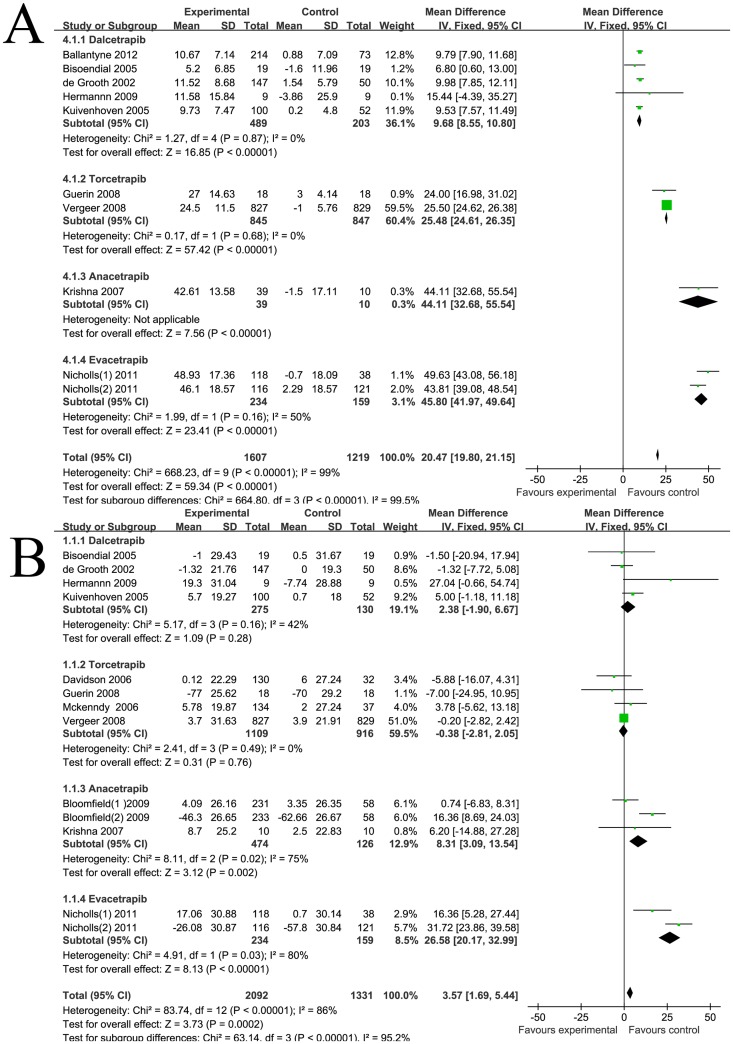
Forest plots depicting the effect of CETP inhibitors on HDL-c and TC (grouped by different CETP inhibitors) A: HDL-c; B: TC.

**Figure 2:**
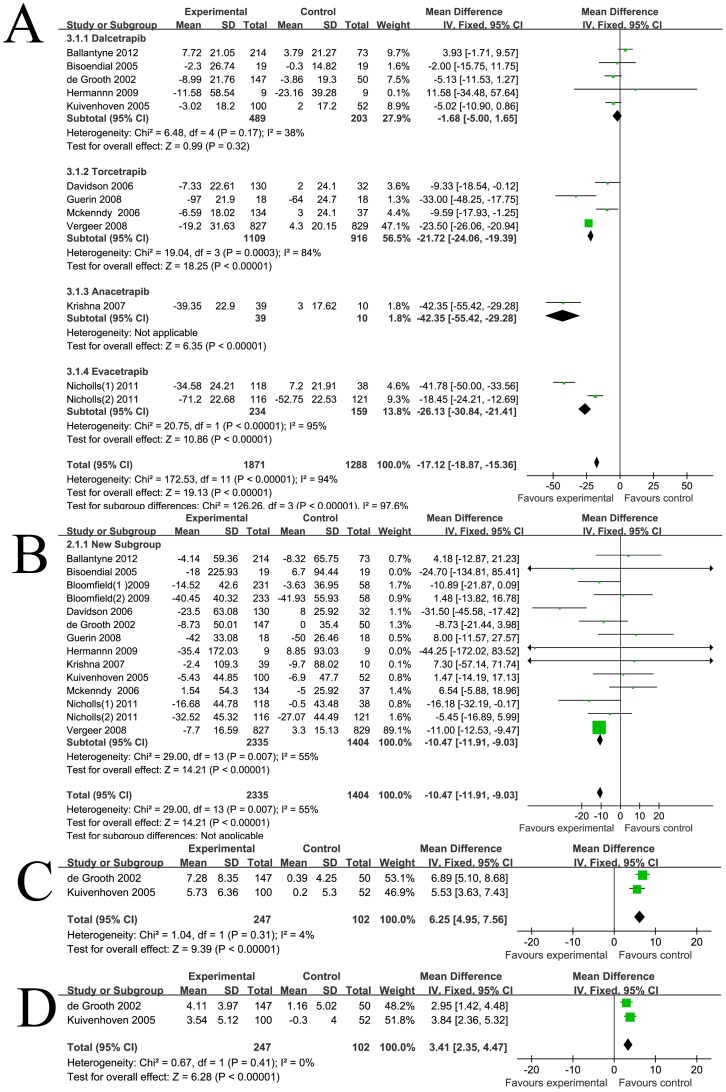
Forest plots depicting the effect of CETP inhibitors on lipid parameters (grouped by different CETP inhibitors) A: LDL-c; B: TG; C: HDL_2_; D: HDL_3_.

**Figure 3:**
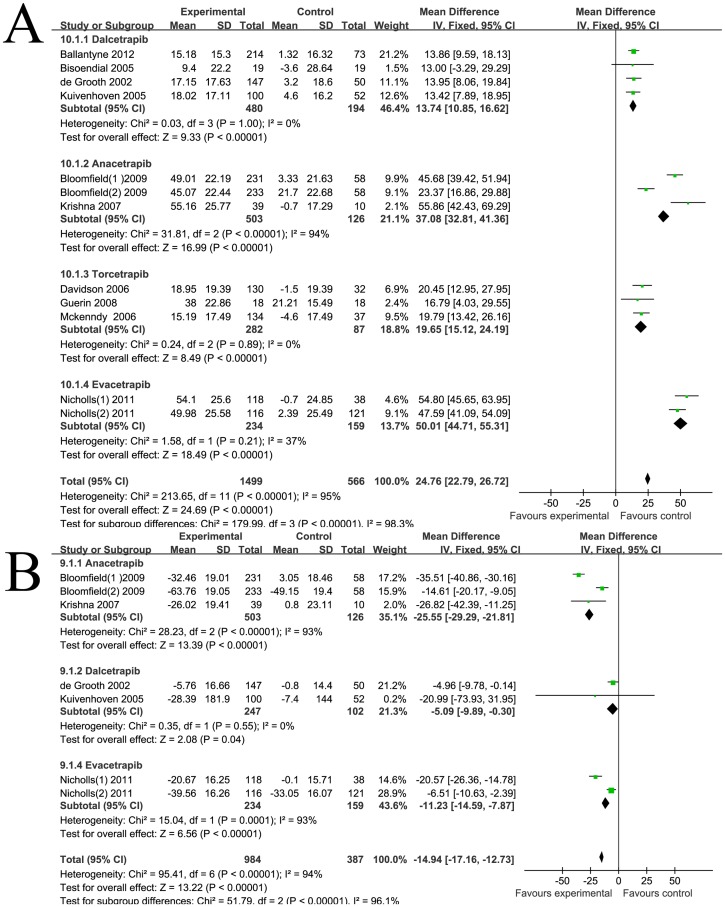
Forest plots depicting the effect of CETP inhibitors on apolipoproteins (grouped by different CETP inhibitors) A: apoAI; B: apoB 100.

As shown in **Figure 1**, the weighted mean net change in HDL-c was 20.47 mg/dl (95% CI [19.8 to 21.15]). Corresponding changes in TC, LDL-c and TG were 3.57 mg/dl (95% CI [1.69 to 5.44]) (**Figure 1**), −17.12 mg/dl (95% CI [−18.87 to −15.36]) (**Figure 2**), −10.47 mg/dl (95% CI [−11.91 to −9.03]) (**Figure 2**) respectively. Significant statistical heterogeneity was observed in HDL-c, TC and LDL-c analysis, the I^2^ were 99%, 85%, 94% respectively. Subgroup analysis was performed comparing the factors listed in the methods. We found that most of the heterogeneity was ascribed to clinical heterogeneity, as different CETP inhibitors have varying lipid modifying effects. The weighted mean changes of HDL-c were: 9.68 mg/dl (95% CI [8.55 to 10.8]) for dalcetrapib, 25.48 mg/dl (95% CI [24.61 to 26.35]) for torcetrapib, 44.11 mg/dl (95% CI [32.68 to 55.54]) for anacetrapib, and 45.8 mg/dl (95% CI [41.97 to 49.64]) for evacetrapib. The discrepancy, judged by age, medicine dosage, treatment duration, dyslipidemia types, mono-therapy or co-administration with statins, baseline lipid levels and study quality, had minor effects on statistical heterogeneity. Sensitivity analysis revealed that Bloomfield's [23] and Nicholls' [25] co-administration therapy group had a major influence on TC and LDL-c levels, which might be due to their different study design. Bloomfield's [23] and Nicholls' [25] studies did not have the statin run-in period, all medicines, including statins and CETP inhibitors, went into the experiments immediately. Therefore, part of the lowering levels of TC or LDL-c might be ascribed to the effects of statin instead of CETP inhibitor. HDL is heterogeneous in particle size, chemical composition and physiological function and represents different stages of dynamic remodeling occurring in the plasma. HDL can be divided into the larger HDL_2_ subclasses and smaller HDL_3_ subclasses by ultracentrifugation [30]. HDL_2_ is more active in the anti-atherosclerosis process and previous studies have proven that larger HDL subclasses exhibit a stronger affinity capability to the cholesterol efflux receptors [31]. HDL subclasses vary in different disease status, and provide further insight into the atherosclerosis risk stratification [32]. Two studies [16]–[17]reported the HDL subclass concentration detected by ultracentrifugation, as shown in **Figure 2**, the net change in HDL_2_ and HDL_3_ after CETP inhibitors treatment were 6.25 mg/dl (95% CI [4.95 to 7.56]) and 3.41 mg/dl (95% CI [2.35 to 4.47]) respectively. These results demonstrate that in adding to the beneficial effects on absolute lipid levels, CETP inhibitors can affect the HDL to a larger degree and to more atherosclerotic-protective subspecies. HDL is the major apoA-I containing lipoproteins, and apoB100 concentrations also parallel with the LDL-c and atherosclerotic capabilities. As shown in **Figure 3**, the pooled mean change in apoA-I and apoB-100 concentration was 24.76 mg/dl (95% CI [22.79 to 26.72]) and −14.94 mg/dl (95% CI [−17.16 to −12.73]) respectively. Subgroup analysis also confirmed that different CETP inhibitors exhibit unique apolipoprotein modifying effects. Sensitivity analysis revealed that Bloomfield's [23] and Nicholls' [25] co-administration therapy group had a major influence on apolipoprotein levels which might also be due to the unique study designs.

### The safety and tolerability outcomes

**Figure 4:**
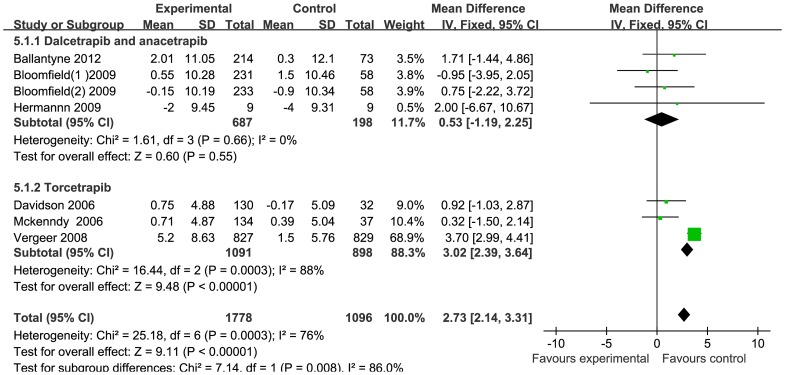
Forest plots depicting the CETP inhibitors on systolic blood pressure.

**Figure 5:**
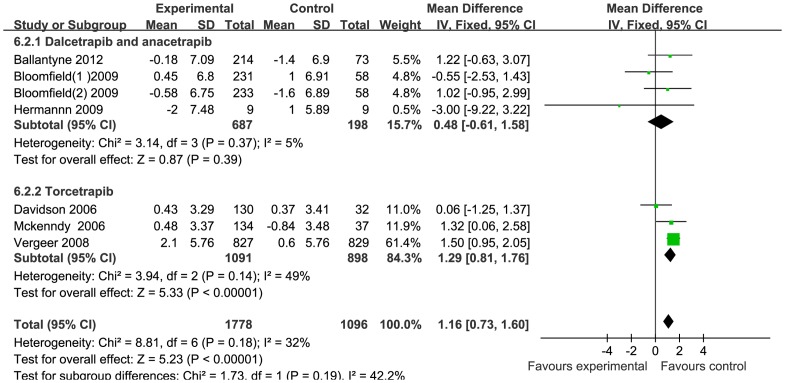
Forest plots depicting the CETP inhibitors on diastolic blood pressure.

Overall, 337 out of 1471 patients receiving CETP inhibitors versus 132 out of 530 patients receiving placebo or statins mono-therapy, experienced medicine-related adverse effects (RR:0.93, 95% CI [0.73 to 1.2]) (**Figure S3**). Most of the drug-related adverse effects were mild or moderate in intensity, with headache, fecal abnormalities, diarrhea and infection as the most frequently reported adverse effects. Subject withdrawal, due to the drug, tended to be higher but was not statistically significant between the treatment and control groups (RR: 1.92, 95% CI [0.98 to 3.75]) (**Figure S4**). Three subjects with hepato-toxicity were reported in the treatment group versus one in the control group (RR: 0.66, 95% CI [0.16 to 2.72]) (**Figure S5**). Musculoskeletal injury events were similar in both treatment and control groups (RR: 0.81, 95% CI [0.24 to 2.74]) (**Figure S6**). Six studies reported little change on the systolic and diastolic blood pressures. The pooled mean change of SBP and DBP were 2.73 mmHg (95% CI [2.14 to 3.31]) and 1.16 mmHg (95% CI [0.73 to 1.6]) respectively (**Figures 4** and 5). Further study using subgroup analysis revealed that the blood pressure changes were ascribed to the effect of torcetrapib, which might activate the rennin-angiotensin-aldosterone system (RAAS), hence increasing the blood pressure via a molecularly-specific way. The other 3 CETP inhibitors, including anacetrapib, dalcetrapib and evacetrapib, had no effect on blood pressure.

## Discussion

We performed a meta-analysis study to determine the efficacy and safety of CETP inhibitors in treating patients with dyslipidemia. The main findings showed that CETP inhibitors exhibit a significant increase in HDL-c and apoAI levels and a decrease in TG, LDL-c, apoB-100 to a small extent irrespective of dyslipidemia types. We also found that CETP inhibitors not only increased the absolute HDL-c levels, but also changed the HDL to larger and more atherosclerotic-protective HDL subspecies. CETP inhibitors exhibited strong lipid modifying effects when co-administered with statins. The rate of adverse effects was not statistically significant between the treatment and control groups. Most of the treatment associated adverse effects were mild and tolerable. CETP inhibitors alone or co-administered with statins did not increase the risk of hepato-toxicity or musculoskeletal injury. A slight increase of SBP and DBP was also observed in this study.

In our meta-analysis, we found that different CETP inhibitors have distinct lipid modifying effects. Evacetrapib seems to be the most effective agent in increasing the HDL-c, followed by anacetorpib, torcetrapib and dalcetrapib. The discrepancies of lipid modifying effects among different CETP inhibitors are largely attributed to the differences in molecular structures. Pharmacological studies revealed that dalcetrapib binds to CETP through the formation of a covalent disulfide bond at its 13^th^ amino acid residue, inducing conformational changes in the protein. Torcetrapib and anacetrapib induce a non-productive complex between CETP and HDL, hence blocking CETP's lipid transfer functions [33]. Evacetrapib is a novel benzazepine-based CETP inhibitor, the CETP inhibitory mechanism remains to be elucidated, but Evacetrapib is more efficient in inhibiting CETP activities. The concentration of Evacetrapib causing half-maximum inhibition of CETP activity was 5.5 nM in vitro analysis, compared to 25.2 nM for torcetrapib and 21.5 nM for anacetrapib [34]. A slight increase in SBP and DBP were observed in patients receiving torcetrapib therapy subgroup. However, we did not find any other similar effects in the other CETP inhibitors, indicating that CETP inhibition per se might not be the cause of the elevated blood pressure. Although the cause of the off-target toxicity needs further investigation, some studies from the animal and cell models revealed that torcetrapib can induce the synthesis of aldosterone and cortisol in a molecularly-specific way [35]. Torcetrapib also induces a sustained impairment of endothelial function and decrease nitric oxide release, stimulate aldosterone secretion as well as vascular reactive oxygen species and endothelin production [36]–[37]. The blood pressure elevating effects of torcetrapib exert a profound influence on CETP inhibitors studies, as in RADIANCE and ILLUSTRATE (Investigating Lipid Level management Using Coronary Ultrasound to Assess Reduction of Atherosclerosis by CETP inhibition and HDL Elevation) study, torcetrapib failed to ameliorate carotid IMT progression and increased the cause of mortality partly due to the elevated blood pressure [38]. Hence, CETP inhibitors without blood pressure elevating off-target toxicity are imperative in the development of novel CETP inhibitors.

HDL is emerging as a novel target for lipid modifying therapy. Although a series of epidemiological studies have observed an inverse relationship between cardiovascular mortality and HDL-c, the beneficial effects of raising HDL-c by the use of treatments with currently available drugs (such as niacin and fibrates) are obscure [39]. Our meta-analysis revealed that CETP inhibitors treatment received satisfactory lipid modifying effects with good safety in patients with dyslipdiemia. Recent meta-analysis revealed that a change of an SD increase of (13.12 mg/dl) in mean change of HDL-c resulting from lipid modifying therapy was associated with a 26% reduction in the risk of cardiovascular death [40]. The main concern of dyslipidemia is the risk of atherosclerosis and associated CVD. In our meta-analysis, only Vergeer's [21] study had a 2-year long treatment duration to evaluate the progression of carotid IMT progression as detected by carotid ultrasonography. In Vergeer's [22] study, despite significant improvement of lipid profiles, the elevated HDL-c failed to prevent the progression of carotid IMT. Recent studies have demonstrated that endogenous low CETP plasma levels constitute an independent risk factor for all-cause and CV mortality, thus indicating that CETP displays anti-atherogenic properties which need to be preserved [41]. Moreover, besides the effect on blood pressure, the effects of individual CETP inhibitors on HDL-c are different and our meta-analysis found that the mean change in HDL-c is heterogeneous among different CETP inhibitors. Evacetrapib and Anacetrapib raise HDL-c more efficiently than dalcetrapib and torcetrapib. The results of dal-OUTCOMES study were published recently. This study enrolled 15,871 patients and evaluated the efficacy and safety of dalcetrapib in reducing mortality and morbidity due to acute coronary syndrome. Dalcetrapib increased HDL-c levels, but failed to reduce the risk of recurrent cardiovascular events [42]. Hopefully, large multi-center randomized control studies of anacetrapib and evacetrapib will provide more evidence to provide to the CETP inhibitor studies.

In conclusion, CETP inhibitors exert excellent effects on the lipid parameters in patients with dyslipidemia even in combination with statin therapy. Given the fact that HDL-c is inversely correlated with CVD mortality, CETP inhibitors could potentially be another novel therapeutic option for CVD treatment.

## Supporting Information

Figure S1 Flow chart of trails.
(TIF)

Figure S2 Risk of bias graph: review authors' judgements about each risk of bias item presented as percentages across all included studies.
(TIF)

Figure S3 Forest plots depicting the treatment associated adverse events.
(TIF)

Figure S4 Forest plots depicting the treatment associated withdrawal.
(TIF)

Figure S5 Forest plots depicting the CETP inhibitors on hepato-toxicity.
(TIF)

Figure S6 Forest plots depicting the CETP inhibitors on muscle-skeletal injury.
(TIF)

Checklist S1 PRISMA Checklist.
(DOC)
