# Supplementary figures and images for: Cholesteryl Ester Transfer Protein Inhibitors in the Treatment of Dyslipidemia: A Systematic Review and Meta-Analysis
Source: PLoS One. 2013 Oct 28;8(10):e77049. doi: 10.1371/journal.pone.0077049 (PMC3810261; doi:10.1371/journal.pone.0077049)

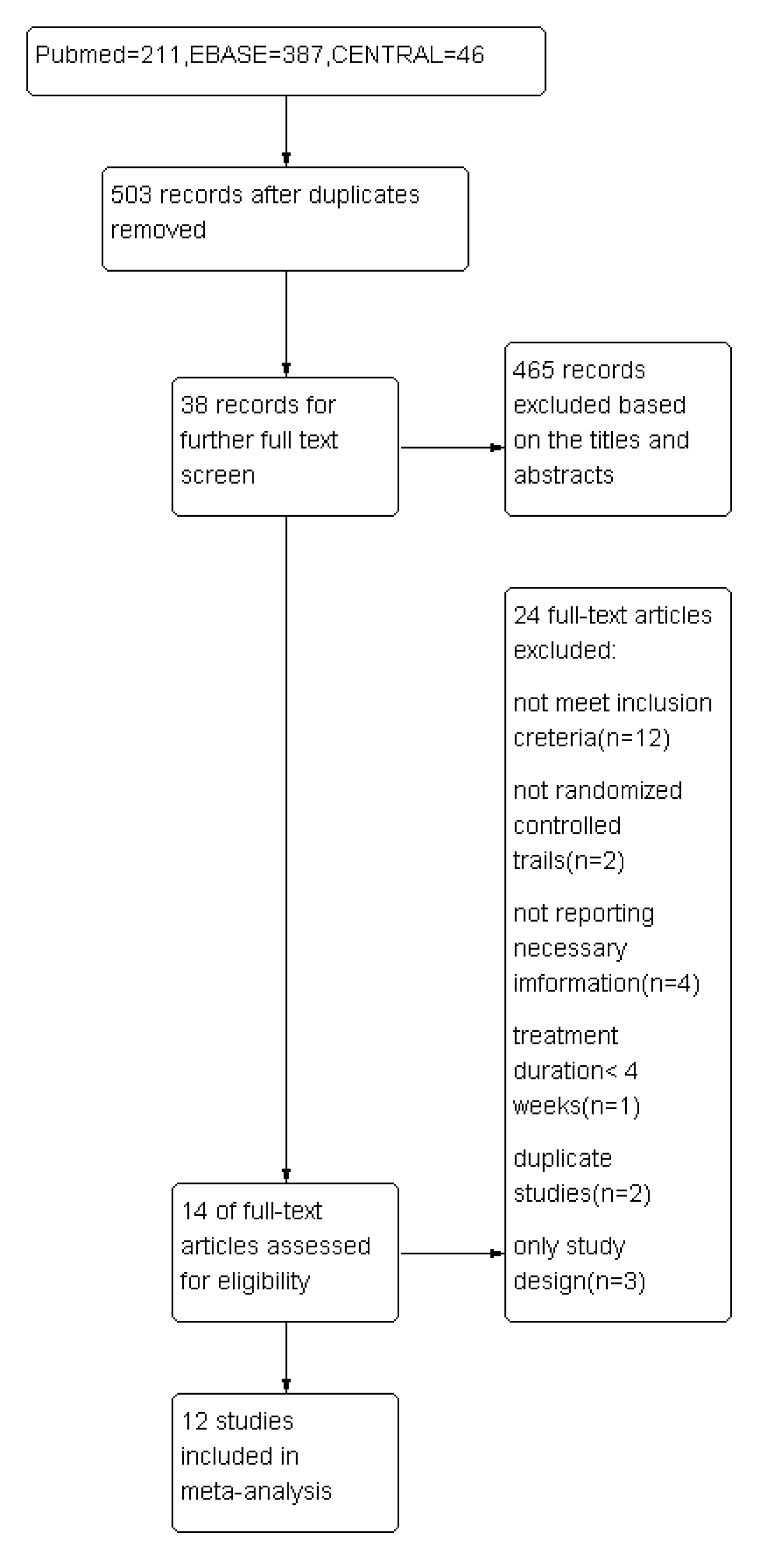

Supplement: Figure S1 — Flow chart of trails. (TIF) [file pone.0077049.s001.tif]

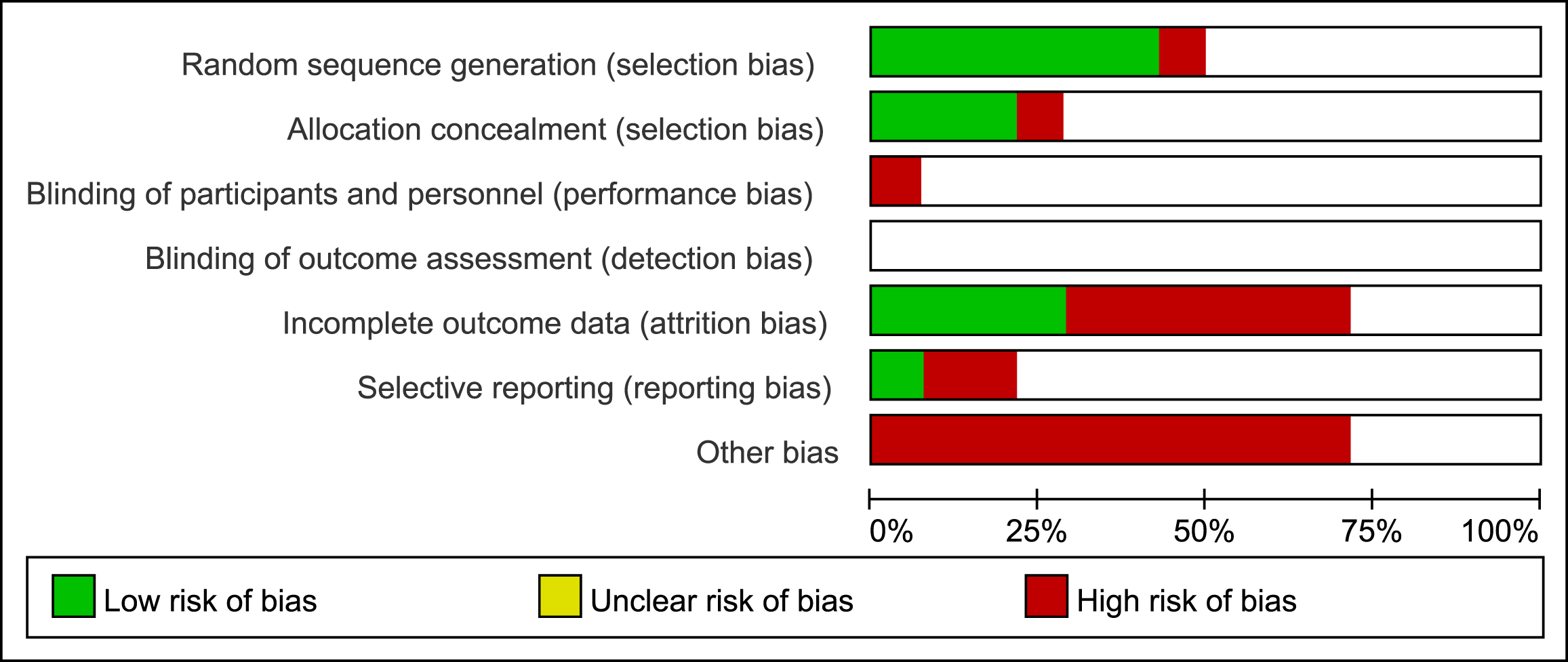

Supplement: Figure S2 — Risk of bias graph: review authors' judgements about each risk of bias item presented as percentages across all included studies. (TIF) [file pone.0077049.s002.tif]

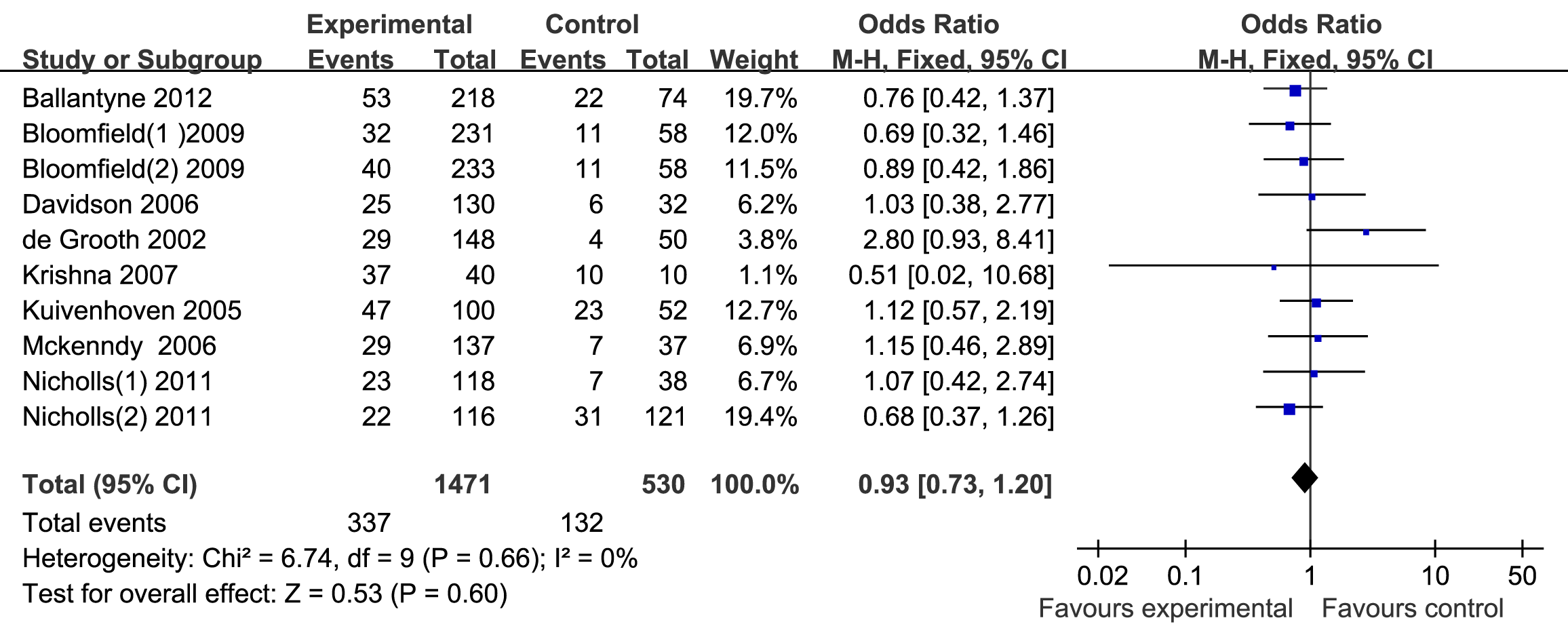

Supplement: Figure S3 — Forest plots depicting the treatment associated adverse events. (TIF) [file pone.0077049.s003.tif]

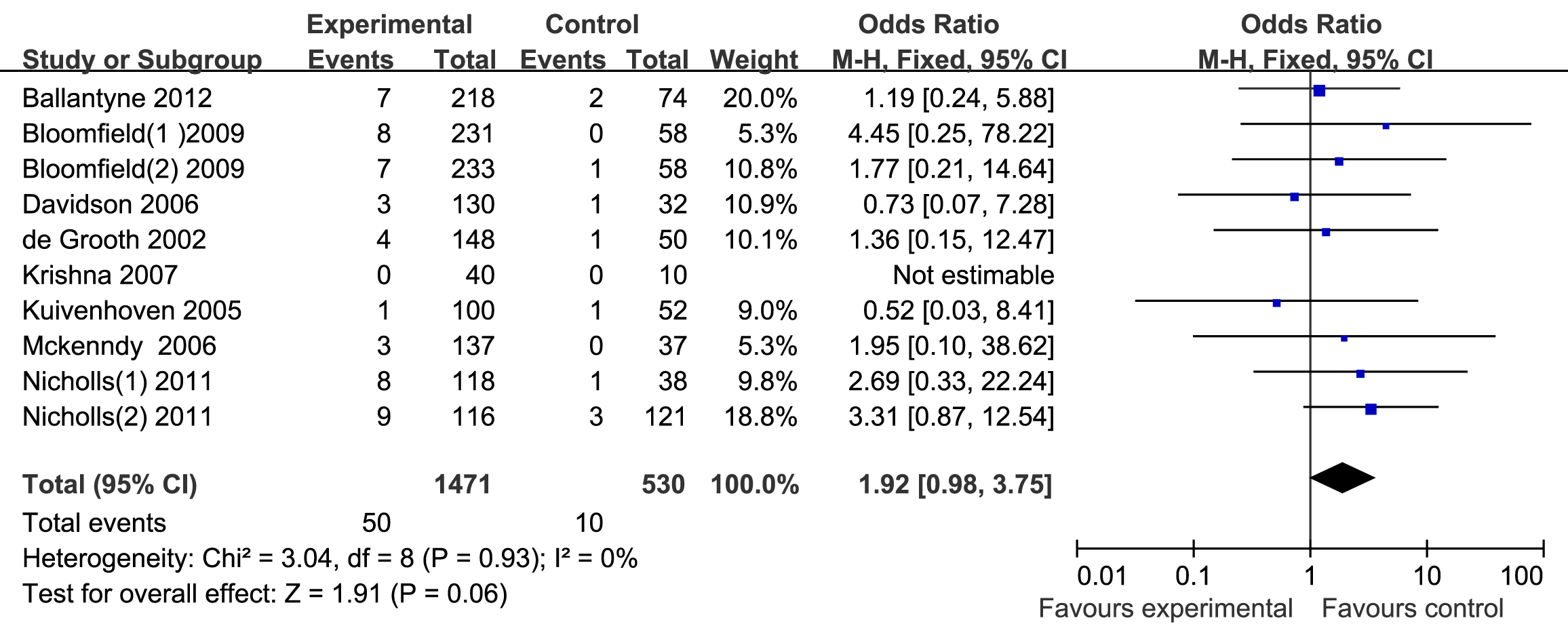

Supplement: Figure S4 — Forest plots depicting the treatment associated withdrawal. (TIF) [file pone.0077049.s004.tif]

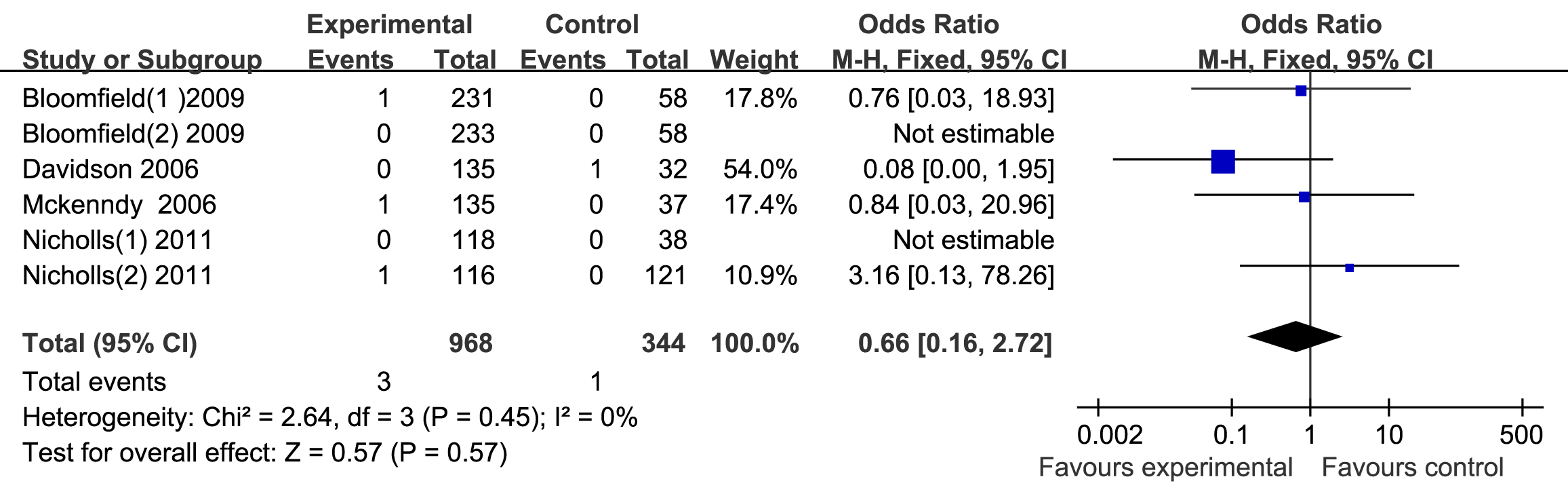

Supplement: Figure S5 — Forest plots depicting the CETP inhibitors on hepato-toxicity. (TIF) [file pone.0077049.s005.tif]

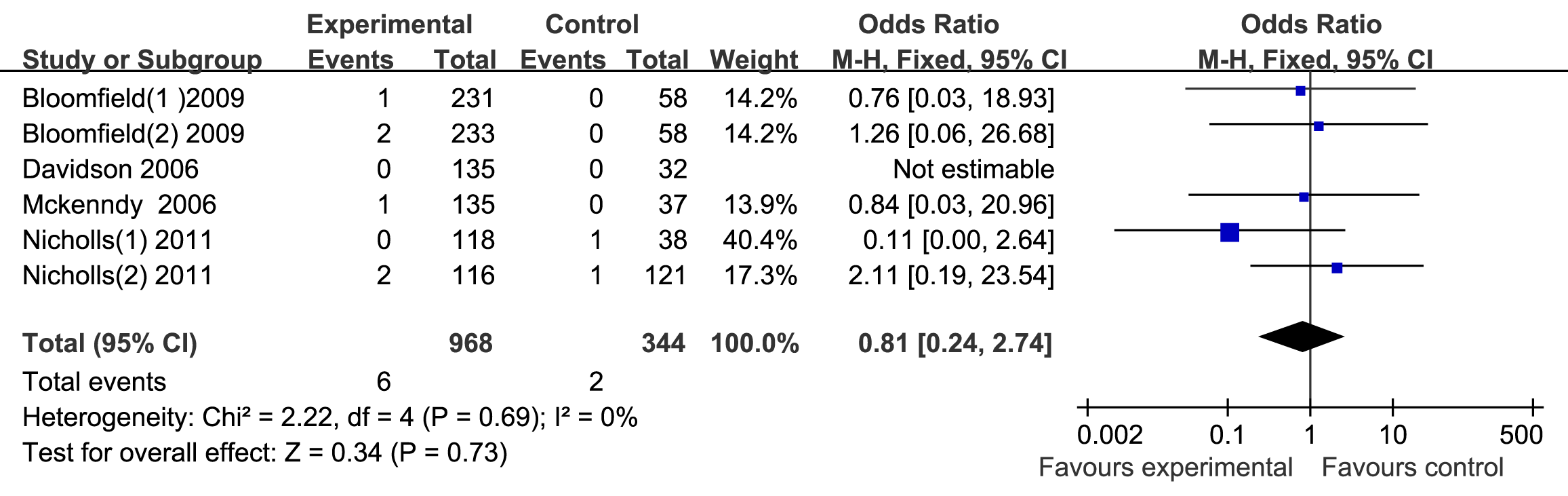

Supplement: Figure S6 — Forest plots depicting the CETP inhibitors on muscle-skeletal injury. (TIF) [file pone.0077049.s006.tif]
